# Hypersonic impact properties of pristine and hybrid single and multi-layer C_3_N and BC_3_ nanosheets

**DOI:** 10.1038/s41598-021-86537-z

**Published:** 2021-04-12

**Authors:** Fatemeh Molaei, Kasra Einalipour Eshkalak, Sadegh Sadeghzadeh, Hossein Siavoshi

**Affiliations:** 1 Mining and Geological Engineering Department, The University of Arizona, Arizona, USA; 2 Qazvin Tarom Copper Company Lab, MSc of Nanotechnology Engineering, School of Advanced Technologies, Iran University of Science and Technology, Tehran, Iran; 3 Nanotechnology Engineering, School of Advanced Technologies, Iran University of Science and Technology, Tehran, Iran

**Keywords:** Nanoscale materials, Surfaces, interfaces and thin films

## Abstract

Carbon, nitrogen, and boron nanostructures are promising ballistic protection materials due to their low density and excellent mechanical properties. In this study, the ballistic properties of C3N and BC3 nanosheets against hypersonic bullets with Mach numbers greater than 6 were studied. The critical perforation conditions, and thus, the intrinsic impact strength of these 2D materials were determined by simulating ballistic curves of C3N and BC3 monolayers. Furthermore, the energy absorption scaling law with different numbers of layers and interlayer spacing was investigated, for homogeneous or hybrid configurations (alternated stacking of C3N and the BC3). Besides, we created a hybrid sheet using van der Waals bonds between two adjacent sheets based on the hypervelocity impacts of fullerene (C60) molecules utilizing molecular dynamics simulation. As a result, since the higher bond energy between N–C compared to B-C, it was shown that C3N nanosheets have higher absorption energy than BC3. In contrast, in lower impact speeds and before penetration, single-layer sheets exhibited almost similar behavior. Our findings also reveal that in hybrid structures, the C3N layers will improve the ballistic properties of BC3. The energy absorption values with a variable number of layers and variable interlayer distance (X = 3.4 Å and 4X = 13.6 Å) are investigated, for homogeneous or hybrid configurations. These results provide a fundamental understanding of ultra-light multilayered armors' design using nanocomposites based on advanced 2D materials. The results can also be used to select and make 2D membranes and allotropes for DNA sequencing and filtration.

## Introduction

Two-dimensional (2D) materials have been able to revolutionize the use of materials in various fields shortly after their discovery. The interface mediating the connection between nanomaterials and other materials or between themselves is the most important factor and place for new features to emerge^1^. One of the most significant 2D materials is graphene. The zero-band characteristic of graphene limits its applications in semiconductors. Two of the known 2D dielectric materials are polyaniline (C_3_N) and BC_3_. Past research shows that C_3_N and BC_3_ are semiconductors with a band-gap of 0.39 eV and 0.54 eV, respectively^2,3^.

Implementing quantum dots could lead to tuning the C_3_N as a semiconductor^4^. Due to the appearance of new properties and their several applications, 2D hybrid materials have been studied by many researchers^5–8^. Recent experimental and theoretical studies focused on the thermal, elastic, electronic, and thermoelectric properties of the C_3_N, BC_3,_ and C_3_N/BC_3_ interface^7,9–15^. Studying the mechanical properties of BC_3_ and C_3_N nanosheets showed that Young's modulus of C_3_N is higher than graphene nanosheet and other 2D materials^16,17^. So, C_3_N and BC_3_ are very good candidates instead of graphene for different applications. The mechanical properties of pristine and defected C_3_N sheets were examined. Increasing the temperature from 200 to 900 K, Young's modulus decreases by 9%^18^. In 2019, Zahedi et al. studied the effect of temperature on the mechanical properties of defected BC_3_ nanosheets. The results of this work were compared with C_3_N and show that the mechanical properties of C_3_N under the same conditions are higher than BC_3_ and the higher values of elastic modulus due to stiffening in C-N bond were compared with C-B bond ones^19^. The mechanical and thermal properties of the hybrid of 2D materials are available due to molecular dynamics simulation^20,21^. Using the MD (spell out on the first mention) technique, in 2013, Zhao et al. examined the mechanical properties of hybrid graphene and hexagonal boron nitride (h-BN) sheet with the concentration of BN, ranging from 0 to 100%. With increasing concentration of BN, Young's modulus of the hybrid sheet diminishes without depending on the distribution of BN. However, adding a small amount of BN to graphene causes a noticeable drop in the strength of the hybrid sheet^20^.

The ballistic properties of 2D materials are of great importance. Protecting structures and devices from the impact of high-energy projectiles is still an open issue for theoretical modeling and applied research. It is also relevant in several technology topics, including materials science and engineering, automotive, aerospace, and defense. Spacecrafts, for instance, are frequently exposed to micrometeoroids and orbital debris hypervelocity collisions (velocities of up to 7–8 km/s). They result in surface degradation, failures onboard instrumentation up to full perforation, and structural damage during operation. In 2017, Signetti et al. studied the ballistic properties of 2D materials due to the high velocity of the collision of a C_60_ molecule using the DFT and FEM simulation methods. The critical penetration energy of graphene membranes and 2D allotropes, including h-BN, was determined as a case study. Besides, the rules of scalability of energy absorption with the variable number of layers and the distance between the layers have been investigated for homogeneous or hybrid configuration^22^. In another study, Rafael et al. studied the scale effect on the ballistic penetration of graphene sheets. In this work, a combination of numerical and analytical modeling has been employed to address this issue. They used the reactive molecular dynamics method and examined ballistic tests for single, double, and triple-layered graphene sheets. Their results showed that the specific penetration energy decreases as the number of layers (N) increases, from ∼15 MJ/kg for N = 1 to ∼0.9 MJ/kg for N = 350, for an impact velocity of 900 m/s^23^. Ballistic tests on 2Dmaterials have also been observed in other works^24,25^.

Although ballistic tests have been performed on 2D materials previously, there have been no reports of the ballistic properties of BC_3_ and C_3_N structures thus far.

Therefore, due to the unique properties of these two structures and potential applications in various industries, as well as the structural similarity with graphene, a more detailed study of the ballistic properties of this type of graphene-like structure is essential. In this study, the ballistic properties of C_3_N and BC_3_ nanosheets against hypersonic bullets with Mach numbers greater than 6 were studied. The critical perforation conditions, and thus, the intrinsic impact strength of these 2D materials were determined by simulating ballistic curves of C_3_N and BC_3_ monolayers. Furthermore, the energy absorption scaling laws with a different number of layers and interlayer spacing was investigated, for homogeneous or hybrid configurations (alternated stacking of C_3_N and the BC_3_). Besides, we created a hybrid sheet using van der Waals bonds between two adjacent sheets based on the hypervelocity impacts of fullerene (C_60_) molecules utilizing molecular dynamics simulation. The findings show the outstanding ballistic properties of the semiconductors BC_3_ and C_3_N in full. These features make them promising designers and also introduce them to the new catalysts for the design of new nanoelectronics and nanoelectromechanical devices.

## Computational methods

A large-scale atomic/molecular massively parallel simulator (LAMMPS) was used for simulation^26^. Image processing and analysis were carried out by OVITO visualization software^27^. The interaction between carbon–nitrogen atoms in C_3_N and carbon-boron in BC_3_, as well as carbon–carbon in the C_60_ molecule, was defined through the Tersoff potential presented by Kinaci et al.^28,29^. However, to investigate the ballistics of these two structures, the optimized potential of interatomic bonds was used in previous reports^30^. In this potential, the relationship between the energy and the displacement of atoms concerning each other is expressed as:

1 $$ U_{ij} = f_{c} \left( {r_{ij} } \right)\left[ {f_{R} \left( {r_{ij} } \right) + b_{ij} f_{A} \left( {r_{ij} } \right)} \right] $$

Function $f_{R} \left( {r_{ij} } \right)$ indicates the repulsion potential of two particles, e.g., in a nucleus-nucleus interaction, and $f_{A} \left( {r_{ij} } \right)$ denotes the attraction potential resulting from valence electrons. $b_{ij}$ is a bonding strength term that depends on the local atomic medium surrounding a specific bond, and it is a decreasing function of atoms rearrangement number. $b_{ij}$ contains all the multi-particle effects of potential. These relations express existing functions in these potentials:

2 $$ f_{R} \left( {r_{ij} } \right){ } = { } - A_{ij} { }e^{{ - {\uplambda }_{ij} r_{ij} }} { },{ }f_{A} \left( {r_{ij} } \right){ } = { } - B_{ij} { }e^{{ - \mu_{ij} r_{ij} }} $$

3 $$  f_{c} \left( {r_{{ij}} } \right) = \left\{ {\begin{array}{*{20}l}    1 \hfill & {r_{{ij}}  < R_{{ij}} } \hfill  \\    {\frac{1}{2} + \frac{1}{2}\cos \left[ {\frac{{\pi \left( {r_{{ij}}  - R_{{ij}} } \right)}}{{S_{{ij}}  - R_{{ij}} }}} \right]} \hfill & {R_{{ij}}  \le r_{{ij}}  \le S_{{ij}} } \hfill  \\    0 \hfill & {r_{{ij}}  < S_{{ij}} } \hfill  \\   \end{array} } \right\} $$

And the required constants are defined as follows:

4 $$ \begin{gathered} b_{ij} = { }X_{ij} \left( {1 + B_{i}^{ni} \xi_{ij}^{ni} } \right)^{{ - 0.5n_{i} }} { ,}\,\,\,\xi_{ij} = { }\mathop \sum \limits_{k \ne i,j} f_{C} \left( {r_{ik} } \right){ }\,\omega_{ik}\, { }g\left( {\theta_{ijk} } \right) \hfill \\ g\left( {\theta_{ijk} } \right) = 1 + \frac{{C_{i}^{2} }}{{d_{i}^{2} }} - \frac{{C_{i}^{2} }}{{d_{i}^{2} + \left( {h_{i} + Cos\theta_{ijk} } \right)^{2} }}{ },\,\,\,\omega_{ik} { } = e^{{\left[ {\mu_{ik}^{3} \left( {r_{{ij - r_{ik} }} } \right)^{3} } \right]}} \hfill \\ \lambda_{ij} = \frac{{\left( {\lambda_{i} + \lambda_{j} } \right)}}{2}{ },{ }\mu_{ij} = \frac{{\left( {\mu_{i} + \mu_{j} } \right)}}{2},\,\,A_{ij} = \sqrt {A_{i} A_{j} } \hfill \\ B_{ij} = \sqrt {B_{i} B_{j} } ,\,\,\,{ }R_{ij} = \sqrt {R_{i} R_{j} } ,\,\,\,S_{ij} = \sqrt {S_{i} S_{j} } \hfill \\ \end{gathered} $$

Indices i, j, and k specify the existing atoms in the ijk bond. rij and rik indicate the lengths of ij and ik bonds, respectively, with θijk being the angle between them. These coefficients have been used concerning the coefficients presented above.

In the study of ballistic properties, for multilayered configurations, which is a non-bonded van der Waals interaction, the Lennard–Jones potential is used. The values of ε and σ can be seen using the following formulas in Table 1.

5 $$ \sigma_{{i{\text{j}}}} = { }\frac{{\sigma_{i} { } + { }\sigma_{j} }}{2},{ }\varepsilon_{ij} = { }\sqrt {\varepsilon_{i} { } \times { }\varepsilon_{j} { }} $$

**Table 1:** Lennard–Jones potential coefficients between different atoms in this study.

Pair	$\sigma$(Å)	$\varepsilon \,\,\, \left( {{\text{eV}}} \right)$
C–N^31^	3.34577013	0.00369113
C–B^31^	3.53419521	0.00596172
N–B^22^	3.409	0.005084
C–C^32^	3.431	0.00455
B–B^22^	3.453	0.004117
N–N^22^	3.365	0.006283

**Figure 1:**
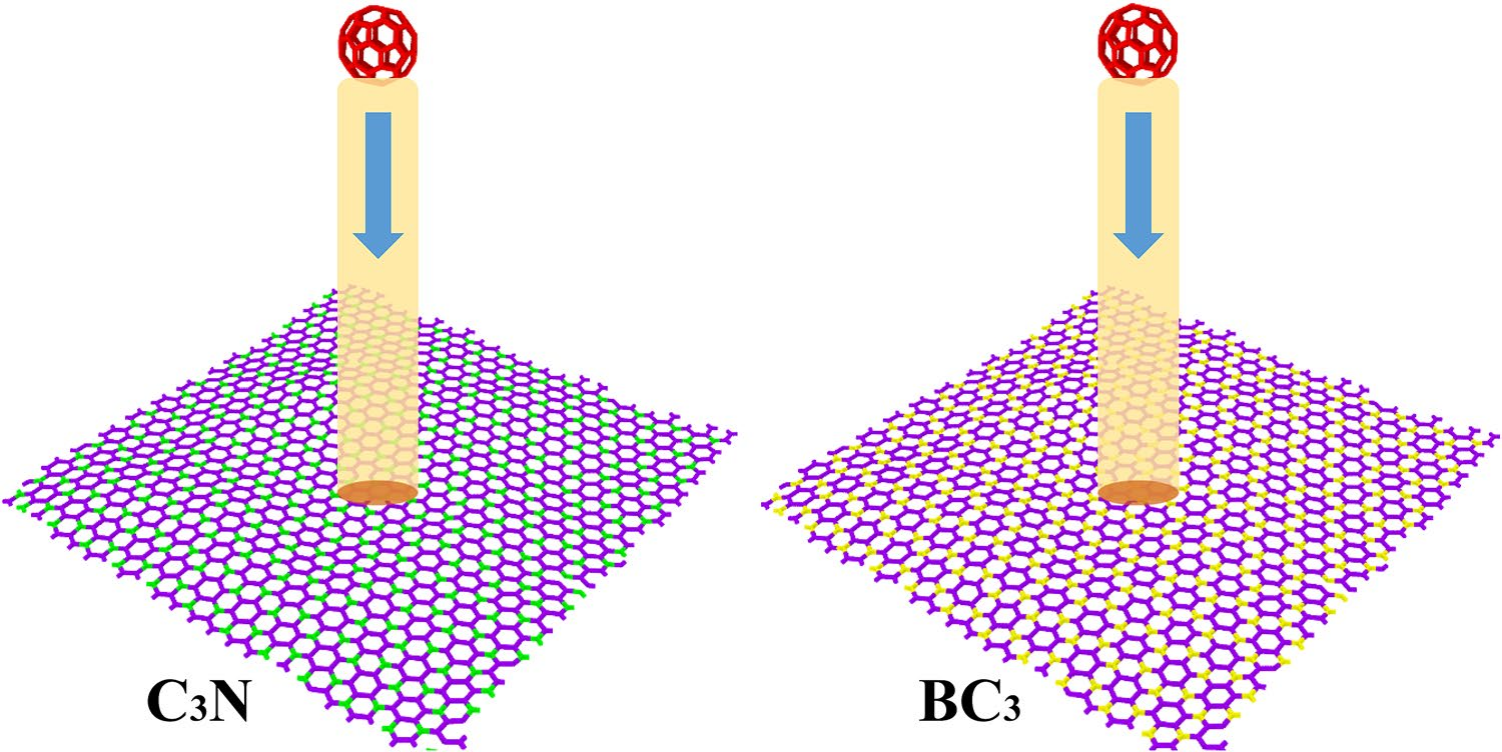
The C_3_N (left) and BC_3_ (right) atomic configuration with a honeycomb structure includes carbon–nitrogen and carbon-boron atoms, respectively. The red bonds show the structure of the fullerene molecule. In their ideal structures, each boron and nitrogen atom is surrounded by three carbon atoms. Carbon, nitrogen, and boron atoms are presented respectively in purple, green, and yellow.

In the present work, the dimensions of the structures 6 × 6 nm^2^ are considered. The total number of atoms present in the simulation is 1404, the share of carbon atoms is 1068 (contains 60 carbon atoms for the fullerene molecule), and the total share of boron and nitrogen atoms in BC_3_ and C_3_N structures is 336 (Fig. 1). Periodic boundary conditions were applied in all three directions. After generating the ensemble of random velocity at 300 K, the system runs to reach out to equilibrium at 300 K under the isothermal-isobaric (NPT) ensemble with the Nose–Hoover thermostat. The time step is 0.25 fs for 50 ps, and the velocity Verlet algorithm was used to integrate the Hamiltonian equations of the determined motion. After equilibrium, the ballistic properties were investigated by throwing a fixed-speed fullerene molecule toward C_3_N and BC_3_. One row of atoms at the boundary of the structure was fixed in both x and y directions. Consequently, the nanosheets maintained their equilibrium when they collided with the fullerene molecule. The distance between the C_60_ molecule and the surface of the nanosheets is considered to be 5 nm.

To better understand the stress distribution in the present work, after computing the stress tensor on each atom, the equivalent stress of a sheet is calculated based on Von Mises stress.

6 $$ {\varvec{\sigma}}_{{{\varvec{V}} - {\varvec{M}}}} = \sqrt {\frac{1}{2}[\left( {{\varvec{\sigma}}_{11} - {\varvec{\sigma}}_{22} } \right) + \left( {{\varvec{\sigma}}_{11} - {\varvec{\sigma}}_{33} } \right) + \left( {{\varvec{\sigma}}_{22} - {\varvec{\sigma}}_{33} } \right) + 6\left( {{\varvec{\sigma}}^{2}_{12} + {\varvec{\sigma}}^{2}_{23} + {\varvec{\sigma}}^{2}_{31} } \right)} $$

That $\sigma_{1,2,3}$ represents the stress in three directions x, y, and z.

## Results and discussion

### Ballistic properties

For vertical stacks of 2D materials, the layers are placed side-by-side with the van der Waals interaction. At the nanoscale, a synergistic interaction occurs between the layers, which is not observed at the micro and macro scales. Several usual layers, less than 10 layers, indicate that a multilayer 2D material has an impact force even higher than its single-layer counterparts. These results provide a basic insight into the design of ultra-light multi-layered armor using nanocomposites based on advanced 2D materials. In a variety of other applications in the electronics field, impact assessment is of significant importance, which can cause unintended and severe shocks during use. Protection with a massive shield is undoubtedly obvious, but it is often impossible because lightness, flexibility, or ergonomics are of particular importance in all of these applications. Therefore, more and more attention has been paid to the development of unconventional nanocomposites with specific toughness and low weight^22^.

**Figure 2:**
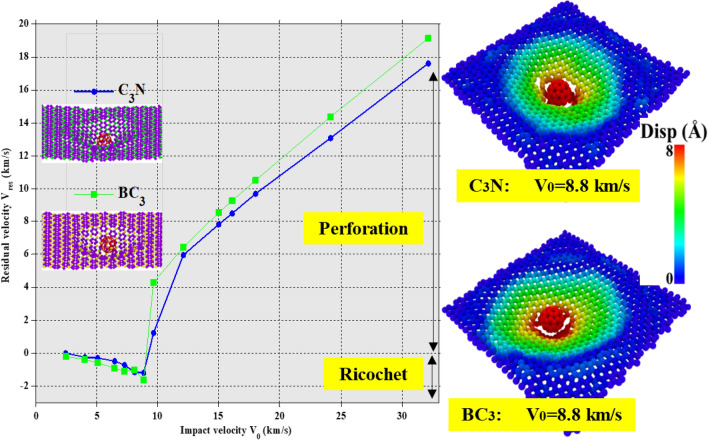
Left panel: Ballistic curves of single-layer C_3_N and BC_3_. The residual velocity V_res_ is referred to as the average velocity of the C_60_ atoms in the Z direction. Right panel: configurations of C_3_N and BC_3_ at the 8.8 km/s initial velocity of C_60_ with MD simulation. The color of each atom indicates its displacement. For visual clarity, the color bar is limited up to 8 Å, and the same color shows all displacements beyond this point.

Thus, in this section, the single-layer and multilayer ballistic properties of C_3_N and BC_3_, including a hybrid of both nanosheets, have been investigated. A C_60_ molecule is thrown at different speeds towards the 2D nanosheets studied in the present work. Drawing the residual velocity curve of the projectile (V_res_) against the initial velocity value of impact (V_0_) is a common method for ballistic analysis to compare the response of different thin armor due to impact (Fig. 2). This diagram is known as the ballistic curve, which easily enables us to differentiate between projectile and penetration regimes so that critical penetration energy can be detected.

In this work, we have considered the initial velocity of the C_60_ molecule from 2.45 to 64.27 km/s in fifteen different values. The projectile's initial and secondary velocity and kinetic energy values can be seen accurately in Table 2. It is clear that C_3_N provides higher penetration velocity and impact energy than single-layer BC_3_. As a result, C_3_N has a lower residual velocity after perforation but shows an almost equal restitution coefficient in the ricochet regime compared to BC_3_.

**Table 2:** Comparison of residual kinetic energy (K_res_) and velocity (V_res_) of monolayer C3N, BC3 using molecular dynamics simulations (present study) and K_res_ and V_res_ of graphene, h-BN using DFT and FEM (previous reports).

	K_0_ (eV)	V_0_ (km/s)	C_3_N		BC_3_	
			K_res_ (eV)	V_res_ (km/s)	K_res_ (eV)	V_res_ (km/s)
Present study	23.3	2.45	0	0	− 0.17	− 0.21
	61.12	4.05	− 0.24	− 0.25	− 0.54	− 0.38
	96.82	5.1	− 0.36	− 0.31	− 1.21	− 0.57
	155.51	6.46	− 0.94	− 0.5	− 3.09	− 0.91
	196.63	7.27	− 1.99	− 0.73	− 4.43	− 1.09
	242.39	8.07	− 5.19	− 1.18	− 3.96	− 1.03
	293.2	8.87	− 5.37	− 1.2	− 10.15	− 1.65
	348.75	9.68	5.64	1.23	68.27	4.28
	543.8	12.08	133.27	5.98	154.08	6.43
	835.13	14.97	228	7.822	273.76	8.571
	955.2	16.1	269.25	8.5	320.24	9.27
	1207.41	18	349.2	9.68	411.63	10.51
	2169.83	24.13	640.5	13.11	769.53	14.37
	3851.88	32.15	1156.98	17.62	1370.92	19.18
	15,393.18	64.27	5190.34	37.32	5962.55	40

			Graphene^22^		h-BN^22^	
Previous reports	33.63	3.0	− 1.30	− 0.059	− 0.03	− 0.09
	59.78	4.0	− 2.47	− 0.81	− 0.14	− 0.19
	93.41	5.0	− 4.30	− 1.07	− 2.00	− 0.73
	134.51	6.0	− 6.43	− 1.31	− 4.83	− 1.14
	183.09	7.0	− 8.41	− 1.50	− 5.37	− 1.20
	209.88	7.5	− 8.07	− 1.47	0.00	0.00
	239.13	8.0	− 7.52	− 1.42	9.42	1.59
	302.65	9.0	− 4.60	− 1.11	51.15	3.70
	336.73	9.5	0.00 0.00	0.00	79.06	4.60
	373.64	10.0	11.15	1.73	113.85	5.52

**Figure 3:**
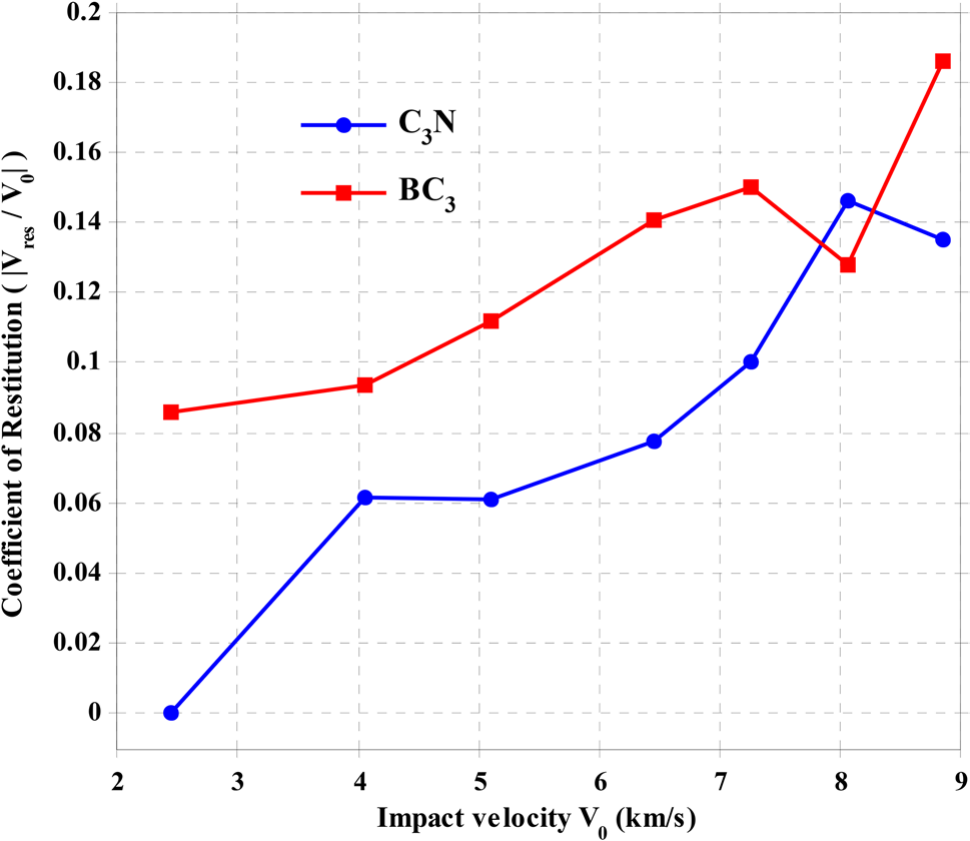
Coefficient of restitution for the collision of a C_60_ molecule along the z-direction.

The restitution coefficient was calculated and plotted versus impact velocity in Fig. 3. As could be observed, when the impact velocity is increased, the restitution coefficient increases gradually. The variation of the restitution coefficient of BC_3_ is more limited concerning the coefficient of C_3_N sheets. As shown in this figure, the restitution coefficient has increased significantly with increasing speed. By increasing the collision speed, which leads to an increase in the relative velocity between the projectile and the sheet, a higher energy rate is obtained, and thus the projectile energy exchange time decreases, which leads to a greater impact on the sheet, resulting in more reaction force per unit time. The return speed increases slightly, which leads to an increase in the restitution coefficient.

From these results, it is clear that BC_3_ sheets absorb more energy from carbon projectiles than C_3_N sheets. From another point of view, perhaps this difference can be related to the natural frequency of oscillations of these two sheets. The natural frequency of the BC_3_ sheet is higher than that of the C_3_N sheet due to its higher flexural strength, and, therefore, it better absorbs high-velocity bullets. This is because when a bullet or projectile approaches its surface, the atoms of these sheets show the possibility of faster displacements due to the higher frequency in response to the presence of the bullet. This issue can be investigated in future studies by studying the free vibrations of these sheets.

**Figure 4:**
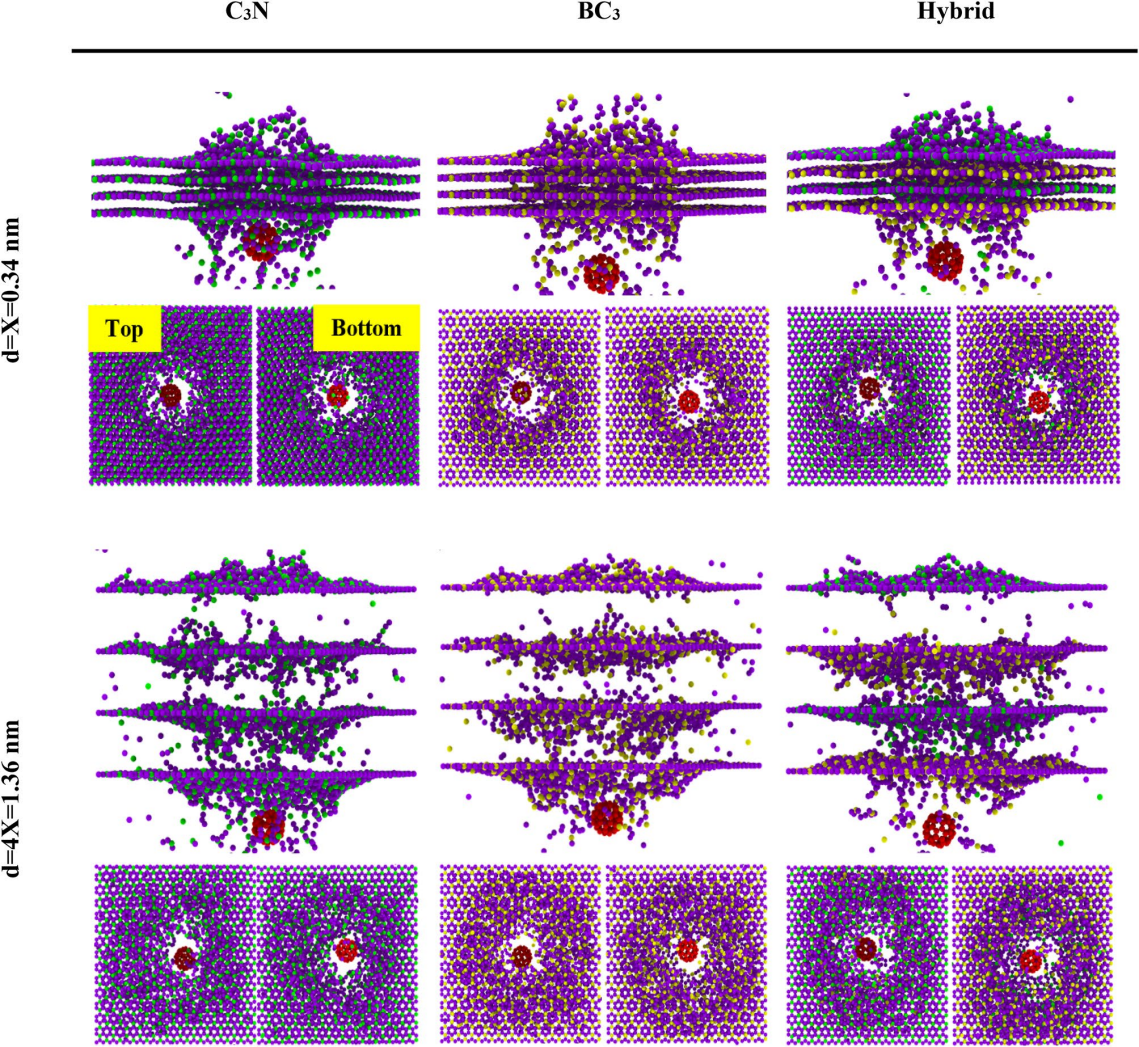
Image of configurations created after the C_60_ impact on four-layer stacks of the C_3_N, BC_3,_ and C_3_N-BC_3_ hybrid nanosheets with K_0_ = 15,393.19 eV for different layer spacing including d = X = 0.34 and d = 4X = 1.36 nm.

The increasing number of layers for the two interlayer distance modes, including d = X = 0.34 nm and d = 4X = 1.36 nm on ballistic properties, has been discussed. Therefore, the C_3_N and BC_3_ multilayer structures and the hybrid of these two sheets are constructed in the form of van der Waals bonds. The C_60_ molecule is thrown towards desired structures with V_0_ = 64.27 km/s and K_0_ = 24.66 × 10^–16^ J. The simulation process in this section for the four-layer model is delineated in both d = X and d = 4X interlayer distances in Fig. 4.

**Figure 5:**
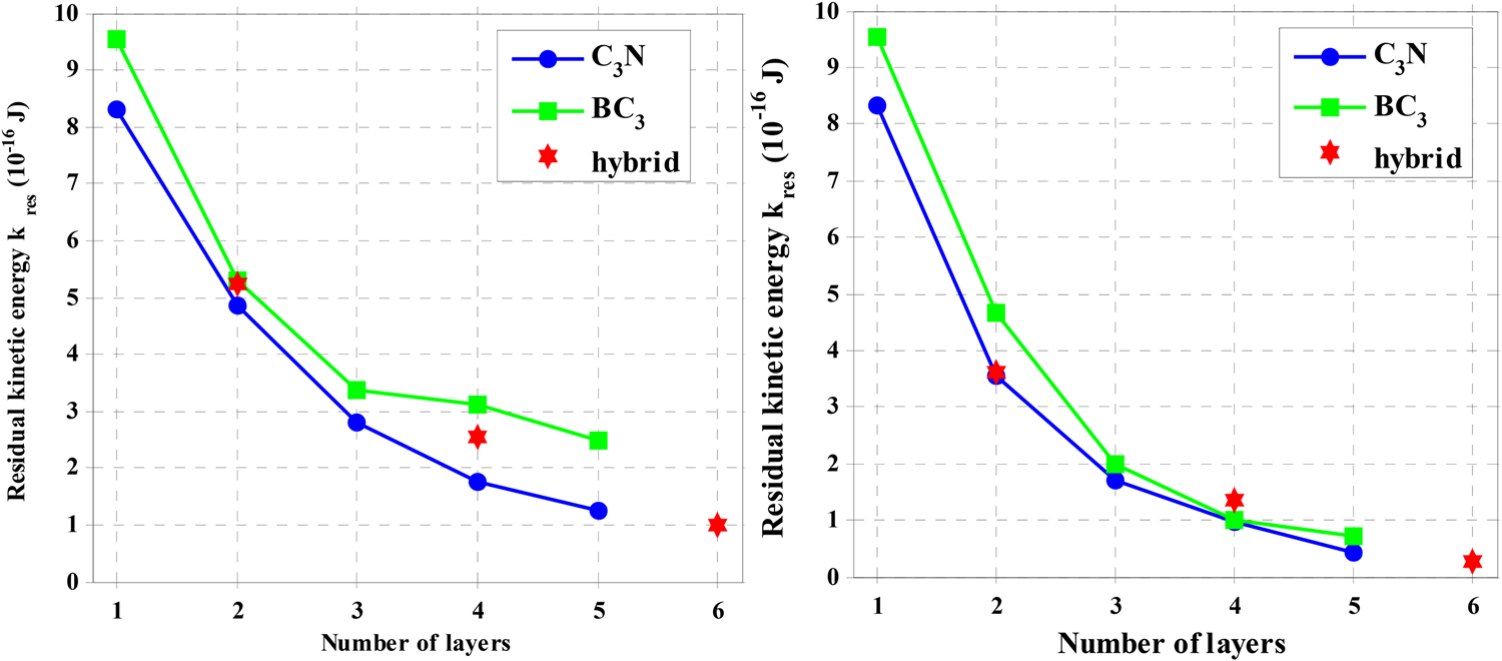
The number of layers’ effect on the residual kinetic energy Kres in the C3N, BC3, and C3N-BC3 hybrid nanosheets with K_0_ = 24.66 × 10–16 J for different layer spacing including d = X = 0.34 and d = 4X = 1.36 nm.

The kinetic energy of all states after impact is shown in Fig. 5. It is entirely evident that C_3_N nanosheets absorb more energy from the C_60_ molecule due to their strong C-N bond. Even when the C_3_N sheet combines with the BC_3_ sheet, their hybrid can increase its ballistic properties and reduce the kinetic energy of fullerenes further. Various mechanisms can be envisioned to increase ballistic properties in this work. The most important reason for the efficiency of C_3_N sheets in comparison with BC_3_ sheets can be attributed to the higher bond energy between N–C compared to B-C (Bonding energies of C–C, N–B, C–N, and C–B are 607, 389, 770 and 448 kcal/mol, respectively). Another reason could be for the increase in Young’s modulus from the C_3_N structure. It is important to note that as the interlayer distance increases from d = X to d = 4X, the amount of kinetic energy decreases significantly. For example, when the interlayer distance is d = 4X, the kinetic energy of C_60_ is 27% lower than when the interlayer distance is d = X. As the distance between layers increases, the removed carbon and nitrogen atoms (for example, in C_3_N) from the first sheet have more space and do not extend along with the C_60_ molecule's motion. When the surface separation from the first layer hits the sides of the second layer, it does not smooth the path of the C_60_ molecule and only causes more damage to the next layers in the whole sheet. However, this does not happen for shorter interlayer distances. As soon as the atoms separate from the first layer, they quickly hit the second layer, and the path of the projectile molecule will be smoother. Therefore, it can be concluded that although increasing the interlayer distance between 2D materials improves the ballistic properties, it also causes irreparable damage to the next layers. Thus, it is not able to withstand excessive pressures for use and application in 2D membranes and purification applications.

Due to the thin nature of the 2D material, these materials may easily deform out-of-plane by arc or wrinkle automatically on the substrates. Although these out-of-plane alterations are very small in size (below the Angstrom scale), they significantly change the effective properties. For example, out-of-plane wrinkles may reduce Young’s modulus of the single-layer, but increase toughness or chemical activity^33^. In the present study, an attempt has been made to remove wrinkles. However, just in the case of C_3_N and BC_3_ combination with a d = 4X layer distance and due to strong van der Waals interaction and prevailing physical and chemical interactions between these two layers, BC_3_ structure towards one layer C_3_Ns is stretched from top to bottom and causes buckling in the system. This stretch can ultimately have little effect on the results.

**Figure 6:**
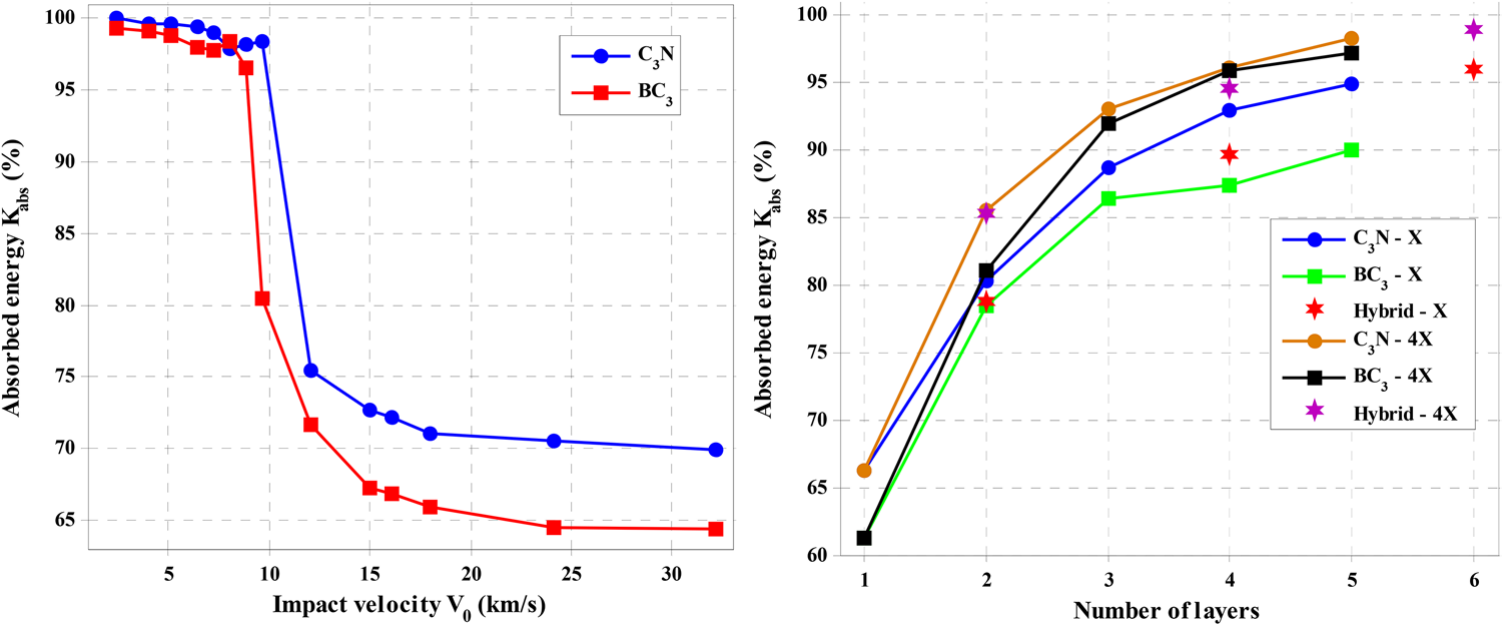
The energy changes adsorbed by the left sheet: increasing impact velocity and right panel: the increasing number of layers for C_3_N, BC_3,_ and their hybrid structures.

In many studies, the single-layer thickness of graphene is assumed to be 0.334 nm, while measurements using an atomic force microscope (AFM) report this value from 0.4 to 1.7 nm^34^. Another important parameter in elastic properties is the amount of energy absorbed by 2D materials, which is obtained by calculating |V_0_^2^ − V_res_^2^|/V_0_^2^. Therefore, Fig. 6 shows absorbed energy changes by increasing impact velocity and increasing the number of layers for C_3_N, BC_3_, and hybrid structures.

**Figure 7:**
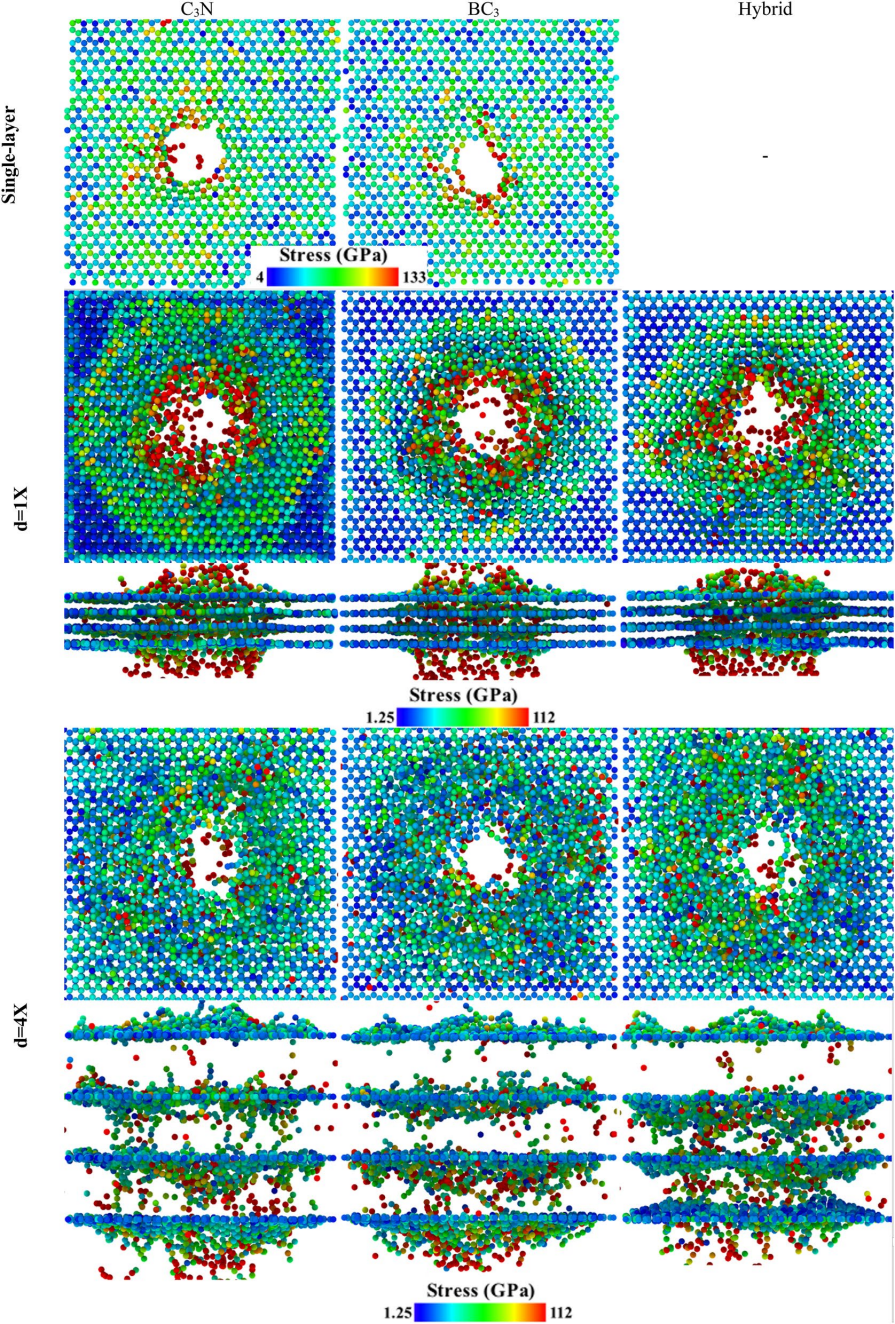
Von Mises stress distribution on a single layer and four-layer stacks of the C_3_N, BC_3,_ and C_3_N-BC_3_ hybrid nanosheets after the C_60_ impact with V_0_ = 64.27 km/s for different layer spacing including X = 0.34 and 4X = 1.36 nm.

To better understand the behavior of stress distribution in different systems after the C_60_ penetration, we obtained the von Mises stress of single-layer and three samples of four-layer structures for d = X and d = 4X layered spacing, which is depicted in Fig. 7. The results show that the stress distribution is different in several modes. The nitrogen and boron atoms do not have the same behavior at different interlayer distances, and this is related to the distribution of stress in the structures. It has been observed that C_3_N has better performance in stress distribution and it distributed maximum stresses uniformly throughout the monolayer sheet. Thus, the stress concentration in this structure has been lower than the others; it will have better mechanical and ballistic properties.

## Conclusions

In this work, we studied the ballistic behavior of single and multi-layered armor C_3_N and BC_3_ and their hybrid, which is exposed to the impact of a high-speed C_60_ molecule by using MD simulation techniques. We determined the critical perforation conditions, and thus, the intrinsic impact strength of these 2D materials, by simulating ballistic curves of C_3_N and BC_3_ monolayers. Furthermore, the energy adsorption scaling law with a variable number of layers and interlayer spacing is investigated, for homogeneous or hybrid configurations. Our results indicated that the speed and energy of the C_60_ molecule dropped sharply after hitting the single layers, which shows the high absorption energy of these two structures. Meanwhile, the absorption energy of C_3_N is higher than BC_3_ and is enhanced the absorption power of BC_3_, even in hybrid systems. In this work, we have introduced the interlayer distance as one of the effective parameters in ballistic properties and showed that when this distance changes from d = X = 0.34 nm to d = 4X = 1.36 nm: for example, in two-layer C_3_N, the kinetic energy of the C_60_ for the 4X interlayer After the collision is calculated to be 27% less than the d = X distance. However, the damage caused by the increase in the interlayer distance for the single layers is also predictable after the C_60_ molecule hits the first sheet. To this end, in the present study, the von Mises stress distribution behavior has been analyzed to create 2D nanoparticles composed of C_3_N and BC_3_. Therefore, the C_3_N structure has a better stress distribution than BC_3_.
